# Supplementary material for: Machine Learning Accelerates Crystallization for Structure Determination
Source: Angew Chem Int Ed Engl. 2026 May 4;65(25):e1218503. doi: 10.1002/anie.1218503 (PMC13266958; doi:10.1002/anie.1218503)

checkCIF/PLATON report

Structure factors have been supplied for datablock(s) lcz012

THIS REPORT IS FOR GUIDANCE ONLY. IF USED AS PART OF A REVIEW PROCEDURE FOR PUBLICATION, IT SHOULD NOT REPLACE THE EXPERTISE OF AN EXPERIENCED CRYSTALLOGRAPHIC REFEREE.

No syntax errors found. CIF dictionary Interpreting this report

Datablock: lcz012

Bond precision:	C-C = 0.0094 A		Wavelength=1.54184
Cell:	a=23.5373(2)	b=8.7893(1)	c=35.3778(3)
	alpha=90	beta=106.676(1)	gamma=90
Temperature:	100 K		

	Calculated	Reported
Volume	7011.02(12)	7011.02(12)
Space group	P 21/n	P 1 21/n 1
Hall group	-P 2yn	-P 2yn
Moiety formula	C26 H17 Ag3 F18 N6 O4	2(C15 H3 Ag3 F18 N6), 2(C11 H14 O4)
Sum formula	C26 H17 Ag3 F18 N6 O4	C52 H34 Ag6 F36 N12 O8
Mr	1143.07	2286.13
Dx,g cm-3	2.166	2.166
Z	8	4
Mu (mm-1)	14.625	14.625
F000	4400.0	4400.0
F000'	4421.34	
h,k,lmax	28,10,42	28,10,42
Nref	12398	12302
Tmin,Tmax	0.194,0.232	0.326,1.000
Tmin'	0.086	

Correction method= # Reported T Limits: Tmin=0.326 Tmax=1.000
AbsCorr = MULTI-SCAN

Data completeness= 0.992   Theta(max)= 66.599

R(reflections)= 0.0451(10320)   wR2(reflections)= 0.1176(12302)

S = 1.057   Npar= 1063

The following ALERTS were generated. Each ALERT has the format
test-name_ALERT_alert-type_alert-level.
Click on the hyperlinks for more details of the test.

Alert level C

```
PLAT041_ALERT_1_C Calc. and Reported SumFormula    Strings  Differ     Please Check
              Calc: C26 H17 Ag3 F18 N6 O4
              Rep.: C52 H34 Ag6 F36 N12 O8
PLAT042_ALERT_1_C Calc. and Reported MoietyFormula Strings  Differ     Please Check
              Calc: C26 H17 Ag3 F18 N6 O4
              Rep.: 2(C15 H3 Ag3 F18 N6), 2(C11 H14 O4)
PLAT213_ALERT_2_C Atom F02Z           has ADP max/min Ratio .....        3.9 prolat
PLAT213_ALERT_2_C Atom F035           has ADP max/min Ratio .....        3.8 prolat
PLAT213_ALERT_2_C Atom F02K           has ADP max/min Ratio .....        3.9 prolat
PLAT213_ALERT_2_C Atom F01U           has ADP max/min Ratio .....        3.4 oblate
PLAT213_ALERT_2_C Atom F022           has ADP max/min Ratio .....        3.8 prolat
PLAT220_ALERT_2_C NonSolvent   Resd 1  F   Ueq(max)/Ueq(min) Range       4.5 Ratio
PLAT220_ALERT_2_C NonSolvent   Resd 2  F   Ueq(max)/Ueq(min) Range       3.3 Ratio
PLAT234_ALERT_4_C Large Hirshfeld Difference F034     --C032     .      0.16 Ang.
PLAT234_ALERT_4_C Large Hirshfeld Difference F02K     --C036     .      0.25 Ang.
PLAT342_ALERT_3_C Low Bond Precision on  C-C Bonds ..............    0.00942 Ang.
PLAT431_ALERT_2_C Short Inter HL..A Contact  F00Y     ..O009     .      2.81 Ang.
                                                     x,y,z  =      1_555 Check
PLAT906_ALERT_3_C Large K Value in the Analysis of Variance ......    12.150 Check
PLAT906_ALERT_3_C Large K Value in the Analysis of Variance ......     2.894 Check
PLAT911_ALERT_3_C Missing FCF Refl Between Thmin & STh/L=    0.595        95 Report
                4  4  0,  26  0  2, -25  0  3,  -7  5  5,   1 10  5,   3 10  5,
                1 10  6,   3 10  6, -15  7  7,  -9  6  7,   3 10  7, -16  8  8,
              -15  7  8, -14  7  8, -13  8  8, -12  8  8, -10  7  8,  -9  7  8,
               -8  7  8,  -7  7  8,   3 10  8,   4 10  8,   8  9  8,   9  9  8,
               22  3  8, -16  8  9, -15  8  9, -14  7  9, -13  7  9, -13  8  9,
                   (   65 More Missing: see the .ckf listing file)
PLAT971_ALERT_2_C Check Calcd Resid. Dens.  1.18Ang From F034          1.71 eA-3
PLAT977_ALERT_2_C Check Negative Difference Density on H01W      .     -0.38 eA-3
```

Alert level G

```
PLAT002_ALERT_2_G Number of Distance or Angle Restraints on AtSite         7 Note
PLAT003_ALERT_2_G Number of Uiso or U(i,j)  Restrained non-H-Atoms         2 Report
PLAT045_ALERT_1_G Calculated and Reported Z Differ by a Factor ...         2 Check
PLAT083_ALERT_2_G SHELXL Second Parameter in WGHT  Unusually Large     23.92 Why ?
PLAT143_ALERT_4_G s.u. on c - Axis Small or Missing .............    0.00030 Ang.
PLAT176_ALERT_4_G The CIF-Embedded .res File Contains SADI Records         3 Report
PLAT186_ALERT_4_G The CIF-Embedded .res File Contains ISOR Records         1 Report
PLAT230_ALERT_2_G Hirshfeld Test Diff for    F02X     --C036     .       7.0 s.u.
PLAT242_ALERT_2_G Low    'MainMol' Ueq as Compared to Neighbors of      C026 Check
PLAT242_ALERT_2_G Low    'MainMol' Ueq as Compared to Neighbors of      C028 Check
```

```
PLAT242_ALERT_2_G Low    'MainMol' Ueq as Compared to Neighbors of       C02W Check
PLAT242_ALERT_2_G Low    'MainMol' Ueq as Compared to Neighbors of       C032 Check
PLAT242_ALERT_2_G Low    'MainMol' Ueq as Compared to Neighbors of       C033 Check
PLAT242_ALERT_2_G Low    'MainMol' Ueq as Compared to Neighbors of       C036 Check
PLAT242_ALERT_2_G Low    'MainMol' Ueq as Compared to Neighbors of       C01Y Check
PLAT242_ALERT_2_G Low    'MainMol' Ueq as Compared to Neighbors of       C024 Check
PLAT242_ALERT_2_G Low    'MainMol' Ueq as Compared to Neighbors of       C02A Check
PLAT242_ALERT_2_G Low    'MainMol' Ueq as Compared to Neighbors of       C02C Check
PLAT242_ALERT_2_G Low    'MainMol' Ueq as Compared to Neighbors of       C02D Check
PLAT242_ALERT_2_G Low    'MainMol' Ueq as Compared to Neighbors of       C02Y Check
PLAT301_ALERT_3_G Main Residue  Disorder .............(Resd    1)          5% Note
PLAT720_ALERT_4_G Number of Unusual/Non-Standard Labels ..........        151 Note
              Ag01    Ag02    Ag03    Ag04    Ag05    Ag06    F007    F008
              O009    F00A    F00B    F00C    F00D    F00E    F00F    F00G
              O00H    O00I    O00J    F00K    F00L    O00M    F00N    F00O
              O00P    F00Q    F00R    F00S    N00T    F00U    F00V    N00W
              F00X    F00Y    O00Z    O010    N011    N012    F013    N014
              F015    N016    F017    N018    N019    N01A    F01B    N01C
              F01D    F01E    N01F    N01G    F01H    F01I    F01J    C01K
              C01L    C01M    C01N    C01O    H01A    H01B    H01C    C01P
              C01Q    C01R    C01S    F01T    C01U    H01U    C01V    C01W
              H01W    C01X    C01Y    C01Z    C020    H020    C021    F022
              C023    H02A    H02B    H02C    C024    C025    H025    C026
              C027    C028    C029    H02J    H02L    H02N    C02A    C02B
              C02C    C02D    C02E    C02F    H02O    H02Q    H02S    C02G
              H02T    C02H    C02I    H02D    H02E    H02F    F02J    C02K
              H02K    C02L    C02M    H02M    C02N    C02O    H02U    C02P
              H02P    C02Q    H02G    H02H    H02I    C02R    H02R    C02S
              C02T    C02U    C02V    H02V    H02W    H02X    C02W    F02X
              C02Y    F02Z    C030    C031    H03A    H03B    H03C    C032
              C033    F034    F035    C036    F02K    F02Y    F01U
PLAT790_ALERT_4_G Centre of Gravity not Within Unit-Cell: Resd.  #          2 Note
              C26 H17 Ag3 F18 N6 O4
PLAT860_ALERT_3_G Number of Least-Squares Restraints ............          15 Note
PLAT909_ALERT_3_G Percentage of I>2sig(I) Data at Theta(Max) Still        75% Note
PLAT910_ALERT_3_G Missing FCF Reflection(s) Below Theta(Min)[Deg]=       2.61 Note
               -1  0  1,
PLAT933_ALERT_2_G Number of HKL-OMIT Records in Embedded .res File         36 Note
                1 10  6,   1 10  5,   7  8 14, -16  8  8,   4  8 14,   1  9 15,
                6  8 15,   9  8 10,   8  8 12,   9  8 11,   5  8 16, -10  7  8,
                7  8 15, -16  8  9,   3 10  6,  -7  7  8,  -2  9 19,  -2  9 18,
               -3  9 20,  -4  9 20,  -9  7  9,   8  8 13,   3  8 15,   6  9 12,
                6  8 14,  -1  9 18,   7  8 16,   6  9 11,  -9  7  8,  -6  9 21,
              -15  8  9,   3 10  5,   0  9 15,   0  9 17, -11  7  9,  13  8 11,
PLAT941_ALERT_3_G Average HKL Measurement Multiplicity ..........         3.0 Low
PLAT969_ALERT_5_G The 'Henn et al.' R-Factor-gap value ..........       2.567 Note
              Predicted wR2: Based on SigI**2  4.58 or SHELX Weight 11.12
PLAT978_ALERT_2_G Number C-C Bonds with Positive Residual Density.          0 Info
```

---

 0 **ALERT level A** = Most likely a serious problem - resolve or explain
 0 **ALERT level B** = A potentially serious problem, consider carefully
18 **ALERT level C** = Check. Ensure it is not caused by an omission or oversight
30 **ALERT level G** = General information/check it is not something unexpected

3 ALERT type 1 CIF construction/syntax error, inconsistent or missing data

```
  28 ALERT type 2 Indicator that the structure model may be wrong or deficient
   9 ALERT type 3 Indicator that the structure quality may be low
   7 ALERT type 4 Improvement, methodology, query or suggestion
   1 ALERT type 5 Informative message, check
```

Validation response form

Please find below a validation response form (VRF) that can be filled in and pasted into your CIF.

```
# start Validation Reply Form
_vrf_PLAT041_lcz012
;
PROBLEM: Calc. and Reported SumFormula    Strings  Differ     Please Check
RESPONSE: ...
;
_vrf_PLAT042_lcz012
;
PROBLEM: Calc. and Reported MoietyFormula Strings  Differ     Please Check
RESPONSE: ...
;
_vrf_PLAT213_lcz012
;
PROBLEM: Atom F02Z            has ADP max/min Ratio .....        3.9 prolat
RESPONSE: ...
;
_vrf_PLAT220_lcz012
;
PROBLEM: NonSolvent   Resd 1  F   Ueq(max)/Ueq(min) Range        4.5 Ratio
RESPONSE: ...
;
_vrf_PLAT234_lcz012
;
PROBLEM: Large Hirshfeld Difference F034     --C032     .       0.16 Ang.
RESPONSE: ...
;
_vrf_PLAT342_lcz012
;
PROBLEM: Low Bond Precision on  C-C Bonds ..............    0.00942 Ang.
RESPONSE: ...
;
_vrf_PLAT431_lcz012
;
PROBLEM: Short Inter HL..A Contact  F00Y     ..O009     .       2.81 Ang.
RESPONSE: ...
;
_vrf_PLAT906_lcz012
;
PROBLEM: Large K Value in the Analysis of Variance ......     12.150 Check
RESPONSE: ...
;
_vrf_PLAT911_lcz012
;
PROBLEM: Missing FCF Refl Between Thmin & STh/L=    0.595         95 Report
RESPONSE: ...
;
```

```
_vrf_PLAT971_lcz012
;
PROBLEM: Check Calcd Resid. Dens.  1.18Ang From F034            1.71 eA-3
RESPONSE: ...
;
_vrf_PLAT977_lcz012
;
PROBLEM: Check Negative Difference Density on H01W      .      -0.38 eA-3
RESPONSE: ...
;
# end Validation Reply Form
```

It is advisable to attempt to resolve as many as possible of the alerts in all categories. Often the minor alerts point to easily fixed oversights, errors and omissions in your CIF or refinement strategy, so attention to these fine details can be worthwhile. It is up to the individual to critically assess their own results and, if necessary, seek expert advice.

PLATON version of 17/09/2025; check.def file version of 17/09/2025

---

duplicate check

No duplication found

Datablock lcz012 - ellipsoid plot

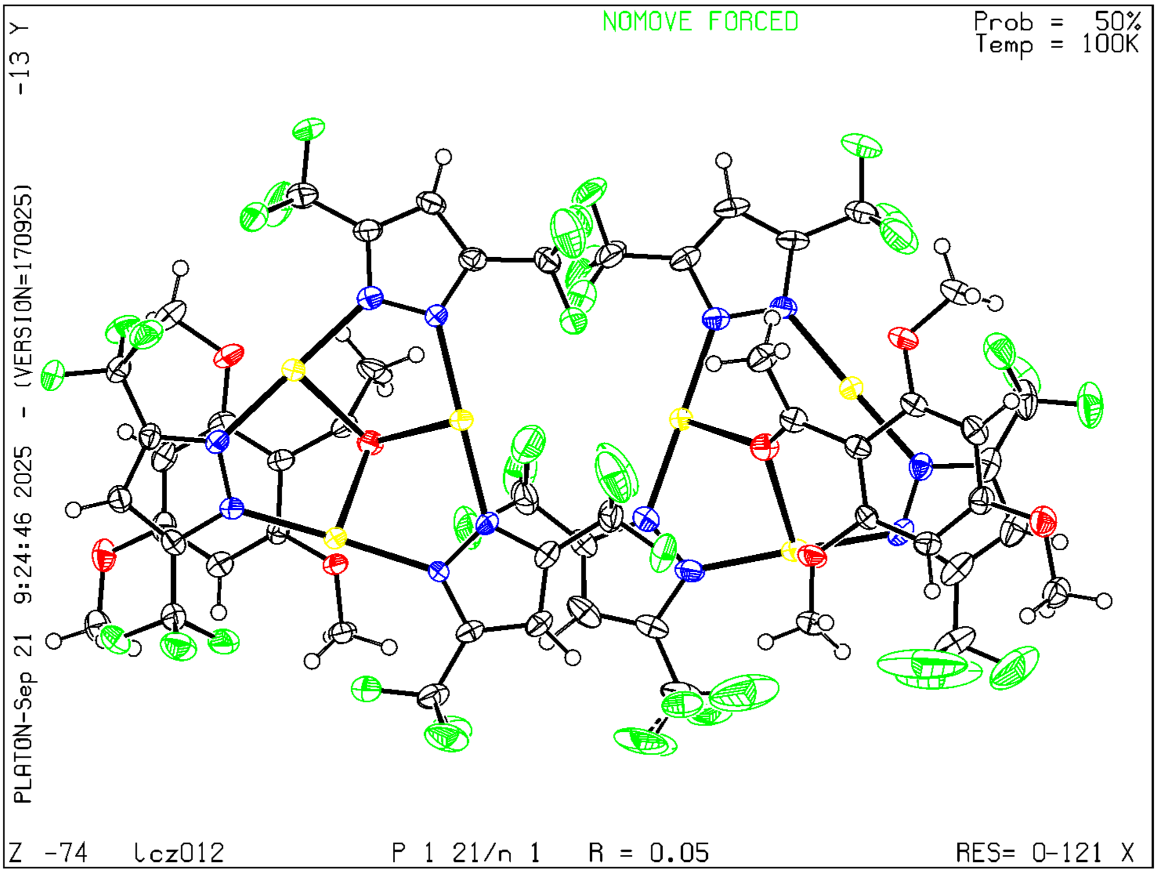

Supplement: Supplementary file 2 — Supporting File 2: anie72462‐sup‐0002‐DataFile.zip. [file ANIE-65-e1218503-s002.zip › supplementary_materials_crystallographic_data submit.zip!/cif_and_checkcif(1-114)/Ag3Pz3_12/Ag3Pz3_12_checkcif.pdf]
